# Symptom-based and self-identified pornography involvement: concordance, discordance, and multidomain correlates

**DOI:** 10.3389/fpsyt.2026.1928379

**Published:** 2026-09-03

**Authors:** Krisztián Széll, Dávid Pócs, Oguz Kelemen, Ádám Tőtös, Edit Paulik, Regina Molnár, Csaba Erdős

**Affiliations:** 1 Faculty of Education and Psychology, Institute of Education, Eötvös Loránd University, Budapest, Hungary; 2 Cardiology Center of Szent-Györgyi Albert Clinical Center, University of Szeged, Szeged, Hungary; 3 Department of Behavioral Sciences, University of Szeged, Albert Szent-Györgyi Medical School, Szeged, Hungary; 4 Department of Public Health, University of Szeged, Albert Szent-Györgyi Medical School, Szeged, Hungary

**Keywords:** concordance, discordance, multidomain correlates, pornography use frequency, pornography-watching disorder, problematic pornography use, self-identified pornography addiction, self-perceived addiction

## Abstract

**Introduction:**

Problematic pornography use (PPU) is increasingly studied, but different self-report operationalizations may not identify the same individuals. In this study, a structured symptom-checklist-based classification of pornography-watching disorder (PWD) was compared with direct self-identified pornography addiction (SIPA). The present study examined the overlap and divergence between these two classifications and compared their behavioral, sociodemographic, psychosexual, and psychosocial correlates.

**Methods:**

Data were drawn from a large Hungarian web-based community survey. A total of 8,705 adults provided information on pornography use frequency (PUF) and SIPA, while multivariable analyses were conducted on a complete-case sample of 8,088 participants. Both classifications were based on self-report. PWD was operationalized using ten DSM-5-inspired symptom indicators, with four or more symptoms indicating PWD, whereas SIPA was measured using a single dichotomous self-identification item. Binary logistic regression models were used to identify factors associated with symptom-defined PWD and SIPA, followed by multinomial logistic regression examining concordant and discordant profiles.

**Results:**

Symptom-defined PWD was identified in 4.8% of participants, whereas 3.1% identified themselves as addicted to pornography. The overlap between the two classifications was moderate (*φ* = .430, *p* <.001). Among participants meeting symptom-based criteria for PWD, 63.7% did not identify themselves as addicted, whereas 43.7% of individuals with SIPA did not meet symptom-based criteria. Symptom-defined PWD was associated with PUF, male sex, greater religiosity, interpersonal difficulties, lower sexual-life satisfaction, earlier exposure to pornography, inadequate sexual education, self-reported sexual disorders, and self-reported paraphilic disorders. In contrast, SIPA was associated primarily with PUF, pornography-related symptom burden, and male sex. Multinomial analyses further showed that individuals who self-identified as addicted despite not meeting symptom-based criteria differed markedly from those with symptom-defined PWD and were distinguished mainly by higher PUF and male sex.

**Discussion:**

Symptom-defined PWD and SIPA represent related but non-equivalent operationalizations of problematic pornography involvement. Whereas the checklist-based PWD classification was associated with a broader range of sociodemographic, psychosexual, and psychosocial characteristics, SIPA was associated primarily with PUF and symptom burden. These findings highlight the importance of distinguishing between structured symptom-checklist-based classification and direct self-identification in both research and clinical assessment.

## 1 Introduction

The rapid expansion of internet access has transformed pornography into one of the most widely consumed forms of online sexual content. Although pornography use is common among adults and is often considered part of normative sexual behavior, concerns have emerged regarding a subgroup of individuals who experience significant difficulties regulating their consumption (1–3). These concerns have contributed to growing scientific interest in problematic pornography use (PPU) and its psychological, relational, and behavioral correlates (4–6).

Current conceptualizations of PPU emphasize impaired control, compulsive engagement, craving, and continued use despite negative consequences. This understanding overlaps with broader clinical discussions of compulsive sexual behavior disorder, which is characterized by persistent failure to control intense and repetitive sexual urges or behaviors resulting in distress or impairment (7, 8). Consistent with addiction-informed frameworks, recent studies suggest that PPU is characterized less by frequency of consumption alone and more by the presence of functional impairment, subjective distress, and loss of control (9–11). Consequently, symptom-based approaches have become increasingly common in empirical research, where problematic involvement is operationalized through indicators reflecting addiction-like symptoms and psychosocial consequences (4, 12, 13).

Importantly, symptom-based classifications represent one operational approach to identifying problematic pornography involvement and should not be equated with formal psychiatric diagnoses.

At the same time, an alternative self-report operationalization has received increasing attention in the literature: direct self-identification as being addicted to pornography. This construct has often been discussed as self-perceived pornography addiction in previous research, whereas the present study uses the term self-identified pornography addiction (SIPA) because it was assessed through a direct self-identification item rather than a multi-item scale. Self-perceived pornography addiction, or SIPA, has been shown to overlap only partially with symptom-based measures of PPU (5, 14, 15). Some individuals describe themselves as addicted despite endorsing relatively few symptom indicators, whereas others endorse multiple symptom indicators but do not consider themselves addicted. This discrepancy has raised important questions regarding the interpretation and comparability of the two approaches. In particular, the pornography problems due to moral incongruence model suggests that self-perceived pornography-related problems may arise not only from behavioral dysregulation but also from conflict between pornography use and personal, moral, or religious values (5, 10, 16).

The distinction between symptom-based classifications and SIPA may have important methodological and potentially clinical implications. A symptom-checklist approach may identify individuals who endorse multiple pornography-related symptoms without adopting an addiction label, whereas direct self-identification may identify individuals who perceive themselves as addicted despite endorsing relatively few symptoms (5, 9, 13). Consequently, prevalence estimates, screening outcomes, and clinical interpretations may vary depending on the operationalization employed. Understanding the factors associated with concordance and discordance between these approaches may therefore contribute to ongoing debates concerning the conceptualization and assessment of PPU.

Despite the growing literature on problematic pornography involvement, the phenomenon has been operationalized in several ways, including symptom-based criteria, validated questionnaire scores, perceived addiction, and self-identification. Although these approaches are often discussed under the same broad conceptual umbrella, relatively few studies have directly compared symptom-based classifications and self-identification within the same sample. It therefore remains unclear to what extent these approaches identify the same individuals and whether they are associated with similar behavioral, sociodemographic, psychosexual, and psychosocial characteristics. Examining these questions may help determine whether observed differences across studies reflect genuine variation in the underlying phenomenon or differences in its operationalization.

Beyond estimating the degree of overlap between operationalizations, examining concordant and discordant classifications may provide additional theoretical insight. Individuals who meet symptom-based criteria but do not identify themselves as addicted, as well as those who self-identify as addicted despite not meeting symptom-based criteria, may represent distinct underlying processes rather than measurement inconsistencies alone. Such discordance may reflect different combinations of pornography use frequency (PUF), perceived dysregulation, religiosity, moral disapproval, moral incongruence, and related appraisal processes that cannot be identified by agreement statistics alone (5, 10, 14, 20, 21). Examining these groups separately may therefore contribute to a more nuanced understanding of problematic pornography involvement.

Previous research has identified several correlates of PPU, including PUF, male gender, younger age, religiosity or moral disapproval, sexual dissatisfaction, interpersonal difficulties, early exposure to pornography, sexual functioning problems, and paraphilic interests or atypical sexual interests (5, 9–11, 15, 17). However, considerably less is known about whether these factors are similarly associated with symptom-based indicators and self-identification as addicted. Most studies have examined these outcomes separately, making it difficult to determine whether they reflect the same underlying phenomenon or partially distinct processes.

In this study, PPU is treated as the overarching construct. PWD represents a structured symptom-checklist-based operationalization of PPU developed in our previous work (18), whereas SIPA captures direct self-identification as addicted to pornography. The original study applied DSM-5-inspired substance use disorder criteria to assess PWD among young adults in Hungary (18, 19). Like SIPA, this classification is based entirely on participants’ self-reports; the distinction lies in the structured aggregation of multiple symptom indicators rather than in greater objectivity or diagnostic status. The term is not intended to imply recognition of PWD as an official DSM-5 or ICD-11 diagnosis. Rather, PWD is used as a checklist-based indicator of problematic pornography involvement, allowing comparison with SIPA.

The primary aim of the present study was to compare two commonly used operationalizations of problematic pornography involvement: a symptom-based classification and direct self-identification as addicted. Using data from a large Hungarian community sample, we examined the degree of overlap between these approaches, compared their behavioral, sociodemographic, psychosexual, and psychosocial correlates, and explored concordant and discordant profiles. Specifically, we first examined factors associated with symptom-defined PWD with logistic regression. Second, we investigated factors associated with SIPA using logistic regression. Finally, we integrated both approaches by creating four concordance profiles based on the joint presence or absence of symptom-defined PWD and SIPA. Using multinomial logistic regression, we examined the behavioral, sociodemographic, psychosexual, and psychosocial characteristics associated with these concordant and discordant profiles.

Based on previous findings, we expected only moderate overlap between the two operational approaches (5, 15). We further expected that the pattern of behavioral, sociodemographic, psychosexual, and psychosocial correlates would differ between the two operationalizations, suggesting that they may capture different aspects of problematic pornography involvement (5, 10). Finally, we anticipated that discordant groups would display distinct multidomain profiles.

Accordingly, the study was guided by four research questions:

RQ1. To what extent do symptom-defined PWD and SIPA overlap?
RQ2. Which factors are associated with symptom-defined PWD?
RQ3. Which factors are associated with SIPA?
RQ4. How do concordant and discordant groups defined by symptom-defined PWD and SIPA differ in their behavioral, sociodemographic, psychosexual, and psychosocial profiles?

## 2 Materials and methods

### 2.1 Participants and procedure

The present study is a secondary analysis of a large Hungarian web-based survey. Data were collected between September and December 2018 through online recruitment, including social media advertisements and the Hungarian-language website of the Albert Szent-Györgyi Medical School, University of Szeged. Participation was voluntary and anonymous, and informed consent was obtained from all participants before questionnaire completion.

The survey assessed pornography consumption patterns, sexual behaviors, sexual health, and psychosocial characteristics. A total of 8,705 respondents aged 18 years or older provided complete information regarding PUF and SIPA and were included in prevalence and concordance analyses. Participants ranged in age from 18 to 89 years (*M* = 23.39, *SD* = 6.32), and 34.2% were male (*n* = 2,978) and 65.8% were female (*n* = 5,727). The descriptive characteristics of the study sample are summarized in **Table 1**.

**Table 1:** Descriptive characteristics of the sample.

Variable	N	%/M (SD)
SIPA	270/8,705	3.1%
Symptom-defined PWD	419/8,700	4.8%
PRS	8,700	0.70 (1.31)
PUF	8,705	—
Never in the last year	1,908	21.9%
Less than monthly	2,094	24.1%
Monthly	1,490	17.1%
One to two times per week	1,700	19.5%
Three to six times per week	1,030	11.8%
One to four times per day	430	4.9%
At least five times per day	53	0.6%
Biological sex	8,705	—
Male	2,978	34.2%
Female	5,727	65.8%
Place of residence	8,698	—
Not in the capital	7,950	91.4%
In the capital	748	8.6%
Age	8,705	23.39 (6.32)
Religiosity	8,669	2.00 (1.98)
Ease of establishing personal relationships	8,705	7.12 (2.16)
Satisfaction with sex life	8,132	6.68 (2.78)
Age at first pornography exposure	8,705	13.92 (3.02)
Adequate sexual education	8,704	—
No	2,565	29.5%
Yes	6,139	70.5%
Sexual disorder	8,705	—
No	7,116	81.7%
Yes	1,589	18.3%
Paraphilic disorder	8,705	—
No	8,566	98.4%
Yes	139	1.6%

For regression analyses, only participants with complete information on all study variables were included, resulting in a final analytical sample of 8,088 respondents. This complete-case approach was used to ensure comparability across the binary and multinomial regression models.

### 2.2 Measures

#### 2.2.1 PUF

Participants reported how frequently they had watched pornography during the previous year. Responses were recorded on an eight-category scale ranging from “Never in the last year” to “More than ten times per day.” Because very few respondents belonged to the two highest-frequency categories, these categories were merged. The resulting seven-category measure was coded as follows: 1 = Never in the last year, 2 = Less than monthly, 3 = Monthly, 4 = One to two times per week, 5 = Three to six times per week, 6 = One to four times per day, and 7 = At least five times per day.

#### 2.2.2 SIPA

SIPA, conceptually related to what previous studies have often termed self-perceived pornography addiction, was assessed through a direct self-report item asking participants whether they considered themselves addicted to pornography: “Do you consider yourself addicted to pornography?” Responses were coded as 0 = No and 1 = Yes. Single-item self-identification measures have been widely used in previous research on perceived pornography addiction and self-perceived dysregulation, particularly in studies examining the role of PUF, religiosity, and moral incongruence in subjective addiction labeling (14, 20, 21). The SIPA item was administered immediately after the ten DSM-5-inspired symptom items within the same survey.

#### 2.2.3 Pornography-related symptom count

Pornography-related symptoms were assessed through ten self-reported dichotomous items adapted from DSM-5 substance use disorder criteria and previously applied in the original PWD study (18, 19). Participants indicated whether each statement had been characteristic of them during the previous year. Responses were coded as 0 = False and 1 = True.

The items assessed impaired control, unsuccessful attempts to reduce pornography use, excessive time investment, craving, functional impairment, interpersonal difficulties, reduction of important activities, hazardous use, continued use despite adverse consequences, and tolerance-like experiences. Responses were summed to create a PRS ranging from 0 to 10, with higher scores indicating a greater number of pornography-related symptoms. Internal consistency of the scale was acceptable in the present sample (Cronbach’s *α* = .71).

#### 2.2.4 Symptom-defined PWD

Following the procedure used in the original study, participants self-reporting four or more of the ten symptom indicators were classified as meeting the study-defined threshold for PWD, whereas those endorsing three or fewer symptoms were classified as not meeting the threshold for PWD (18). This threshold reflects the DSM-5-based logic used in the original operationalization, where substance use disorder criteria were adapted to pornography use to capture a clinically more relevant pattern of problematic involvement than frequency alone (18, 19).

Although the broader literature typically refers to PPU, the term PWD is used when describing this DSM-5-inspired symptom-based classification. Conceptually, however, PWD is treated in the present study as a symptom-defined indicator of problematic pornography involvement, broadly aligned with contemporary approaches emphasizing impaired control, distress, and functional impairment rather than consumption frequency alone (5, 9).

#### 2.2.5 Concordance between symptom-defined PWD and SIPA

To examine the overlap between the checklist-based PWD classification and SIPA, a four-category concordance variable was constructed. The checklist-based dimension was based on the PWD classification described above, whereas the direct self-identification dimension was based on participants’ response to the SIPA item. Both dimensions were derived from self-reported information. The resulting four groups were:

1. Concordant non-problematic group: no symptom-defined PWD and no SIPA;
2. PWD-only group: symptom-defined PWD without SIPA;
3. SIPA-only group: SIPA without symptom-defined PWD;
4. Both PWD and SIPA group: presence of both symptom-defined PWD and SIPA.

This classification was designed to distinguish individuals for whom symptom-defined PWD and SIPA converge from those for whom they diverge. This distinction is consistent with prior research showing that self-perceived pornography addiction and measures of PPU overlap only partially and may reflect partly distinct mechanisms (5, 14, 20, 21).

#### 2.2.6 Sociodemographic characteristics

Biological sex was coded as male or female. Place of residence was categorized as residing in the capital city versus elsewhere in Hungary, coded as 0 = not in the capital and 1 = capital. Age was measured in years. These sociodemographic variables were included based on prior findings. Sex and age have been identified as relevant correlates of pornography use, PWD, and self-perceived pornography-related problems, while place of residence was retained because non-capital residence had previously been associated with symptom-defined PWD in the original study using the same Hungarian dataset (18, 20).

#### 2.2.7 Psychosexual characteristics

Psychosexual variables were selected based on prior findings that identified them as potential correlates of PWD. The original PWD study using the same Hungarian dataset identified PUF, male sex, earlier exposure to pornography, paraphilia, lower sexual life satisfaction, difficulty forming personal relationships, stronger adherence to religious norms, and inadequate sexual education as relevant factors associated with PWD (18). In addition, previous research has linked self-perceived pornography addiction to use frequency, younger age, male gender, religiosity, moral incongruence, and subjective distress (5, 14, 20, 21). Recent large-scale work has further suggested that PUF, avoidance- and stress-reduction motives, moral incongruence, and sexual shame are among the most robust predictors of PPU (22).

Several subjective evaluations were assessed using 10-point rating scales, a format commonly used to capture ordered subjective appraisals. Religiosity was measured on a 10-point scale ranging from 1 = “Do not follow religious norms at all” to 10 = “Fully follow religious norms.” Ease of establishing personal relationships was assessed on a 10-point scale ranging from 1 = “Very difficult” to 10 = “Very easy.” Sexual life satisfaction was measured on a 10-point scale ranging from 1 = “Not satisfied at all” to 10 = “Completely satisfied.” Age at first exposure to pornographic content was recorded in years. Participants also reported whether they had received adequate and sufficient sexual education before their first sexual intercourse; responses were coded as 0 = No and 1 = Yes. Sexual disorder status was determined using DSM-5-based self-report criteria and indicated whether participants self-reported symptoms meeting the study criteria for at least one assessed sexual disorder (19). This variable was coded as 0 = No and 1 = Yes. Paraphilic disorder status was determined using DSM-5-based self-report criteria requiring the simultaneous presence of both Criterion A and Criterion B (19) and was coded dichotomously as 0 = No paraphilic disorder and 1 = Paraphilic disorder present. The sexual disorder status and paraphilic disorder status variables represent self-reported indicators rather than clinically confirmed diagnoses.

### 2.3 Statistical analysis

Descriptive statistics were calculated for all study variables. The prevalence of symptom-defined PWD and SIPA was estimated using the largest available sample for each measure. Concordance between PWD and SIPA was examined using cross-tabulations, chi-square tests, and Cramér’s *V*.

To examine factors associated with symptom-defined PWD and SIPA, two separate binary logistic regression models were estimated. Both models included the same set of behavioral, sociodemographic, psychosexual, and psychosocial variables: PUF, biological sex, age, place of residence, religiosity, ease of establishing personal relationships, adequate sexual education before first sexual intercourse, sexual life satisfaction, age at first exposure to pornography, sexual disorder status, and paraphilic disorder status. PRS was additionally included in the SIPA model to examine whether pornography-related symptom burden was independently associated with self-identification as addicted. PRS was not included in the PWD model because the symptom-defined PWD classification was itself derived from these symptom indicators, and its inclusion would therefore have introduced circularity. Both binary logistic regression analyses were conducted on the subset of participants with complete data across all variables included in the models (N = 8,088).

Differences among the four concordance groups were examined using chi-square tests for categorical variables and Welch’s ANOVA for continuous variables. Welch’s ANOVA was used because the concordance groups differed substantially in size. Effect sizes are reported as eta-squared (η²). Because several continuous or ordinal variables were non-normally distributed, Kruskal–Wallis tests were additionally conducted as robustness checks. These analyses produced the same pattern of statistically significant and non-significant findings as the parametric analyses.

Finally, multinomial logistic regression analysis was conducted to identify factors associated with concordant and discordant profiles of symptom-defined PWD and SIPA. The concordant non-problematic group served as the reference category. Odds ratios (ORs) and 95% confidence intervals (CIs) are reported throughout. The multinomial logistic regression was likewise estimated on the complete-case sample (N = 8,088).

Sensitivity analyses were conducted to evaluate (1) alternative specifications of PUF and (2) the robustness of the findings in a subsample of participants aged 35 years or younger.

All analyses were conducted using IBM SPSS Statistics 31 (IBM Corp., Armonk, NY, USA).

### 2.4 Ethics statement

The study protocol was reviewed and approved by the Regional and Institutional Human Medical Biological Research Ethics Committee of the University of Szeged (Approval No. 161/2017-SZTE). Participation was voluntary, and informed electronic consent was obtained from all participants before completing the questionnaire. The study was conducted in accordance with the ethical principles outlined in the Declaration of Helsinki. All data were collected anonymously and analyzed in anonymized form to protect participants’ privacy and confidentiality.

## 3 Results

### 3.1 Prevalence of symptom-defined PWD and SIPA

SIPA was reported by 270 of the 8,705 participants (3.1%). Among participants with valid symptom data (N = 8,700), 419 (4.8%) met the criteria for symptom-defined PWD (see **Table 1**). The association between SIPA and symptom-defined PWD was moderate in magnitude (*φ* = .430, *p* <.001), indicating moderate agreement between the two operationalizations.

Among participants who met the symptom-based criteria for PWD (*n* = 419), only 36.3% also identified themselves as addicted to pornography, whereas 63.7% did not report such self-identification. Conversely, among participants who considered themselves addicted to pornography (*n* = 270), 43.7% did not meet the symptom-based criteria for PWD.

**Figure 1:**
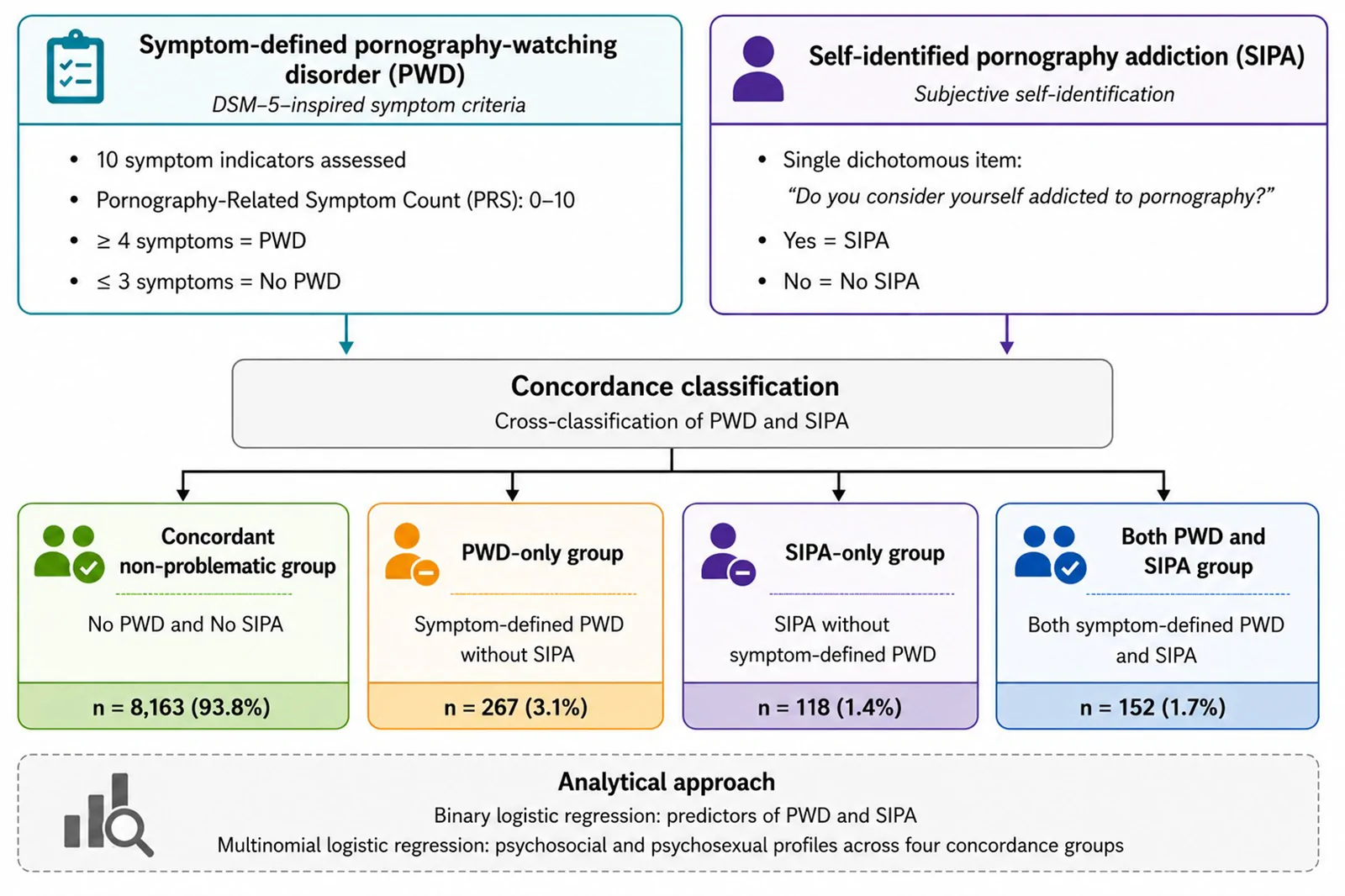
Concordance classification of symptom-defined PWD and SIPA. Percentages are based on participants with valid classification data (N = 8,700).

Based on the combination of symptom-defined PWD and SIPA, four groups were distinguished: (1) concordant non-problematic group (*n* = 8,163, 93.8%), (2) PWD-only group (*n* = 267, 3.1%), (3) SIPA-only group (*n* = 118, 1.4%), and (4) both PWD and SIPA group (*n* = 152, 1.7%) (**Figure 1**).

### 3.2 Factors associated with symptom-defined PWD

A binary logistic regression analysis was conducted to examine factors associated with symptom-defined PWD (**Table 2**). The model was statistically significant, *χ²*(11) = 546.81, *p* <.001, with Nagelkerke *R²* = .220.

**Table 2:** Binary logistic regression models of factors associated with symptom-defined PWD and SIPA.

Variable	PWD OR (95% CI)	p	SIPA OR (95% CI)	p
PRS	—	—	2.10 [1.93, 2.28]	<.001
PUF	2.03 [1.84, 2.24]	<.001	2.14 [1.84, 2.50]	<.001
Biological sex (Male vs Female)	1.52 [1.13, 2.04]	.006	2.47 [1.57, 3.89]	<.001
Place of residence (Capital vs Non-capital)	0.54 [0.32, 0.93]	.026	1.02 [0.52, 2.00]	.952
Age	0.98 [0.96, 1.00]	.081	1.01 [0.99, 1.04]	.336
Religiosity	1.11 [1.05, 1.17]	<.001	0.97 [0.89, 1.06]	.486
Ease of establishing personal relationships	0.93 [0.88, 0.98]	.003	0.99 [0.92, 1.07]	.830
Satisfaction with sex life	0.96 [0.92, 0.99]	.028	1.04 [0.98, 1.11]	.184
Age at first pornography exposure	0.95 [0.91, 0.99]	.020	1.02 [0.95, 1.08]	.656
Adequate sexual education (Present vs Absent)	0.69 [0.54, 0.87]	.002	0.87 [0.61, 1.25]	.454
Sexual disorder (Present vs Absent)	1.86 [1.36, 2.55]	<.001	1.04 [0.61, 1.75]	.898
Paraphilic disorder (Present vs Absent)	3.23 [1.97, 5.28]	<.001	1.72 [0.87, 3.39]	.121

PWD model: Nagelkerke *R²* = .220. SIPA model: Nagelkerke *R²* = .493.

Higher PUF was strongly associated with symptom-defined PWD. Each one-category increase in PUF was associated with approximately doubled odds of meeting the criteria for PWD, *OR* = 2.03, 95% CI [1.84, 2.24], *p* <.001. Men were also significantly more likely than women to meet the criteria for PWD, *OR* = 1.52, 95% CI [1.13, 2.04], *p* = .006. Place of residence was also significantly associated with PWD status, with participants living in the capital showing lower odds of meeting PWD criteria than those living outside the capital (*OR* = 0.54, 95% CI [0.32, 0.93], *p* = .026).

Several psychosocial and psychosexual variables were independently associated with symptom-defined PWD. Higher religiosity was associated with greater odds of PWD, *OR* = 1.11, 95% CI [1.05, 1.17], *p* <.001. In contrast, greater ease of establishing personal relationships, *OR* = 0.93, 95% CI [0.88, 0.98], *p* = .003, and higher sexual-life satisfaction, *OR* = 0.96, 95% CI [0.92, 0.99], *p* = .028, were associated with lower odds of PWD. Earlier exposure to pornography was associated with an increased likelihood of PWD, such that each one-year increase in age at first exposure was associated with a 5% decrease in the odds of meeting PWD criteria, *OR* = 0.95, 95% CI [0.91, 0.99], *p* = .020. Participants reporting inadequate sexual education, sexual disorders, and paraphilic disorders were significantly more likely to meet symptom-based criteria for PWD (see **Table 2**).

Taken together, these findings indicate that symptom-defined PWD was associated not only with PUF but also with a broader range of sociodemographic, psychosexual, and psychosocial characteristics.

### 3.3 Factors associated with SIPA

A second binary logistic regression analysis was conducted to examine factors associated with SIPA using the same behavioral, sociodemographic, psychosexual, and psychosocial variables, with PRS additionally included as a predictor (**Table 2**). The model was statistically significant, *χ²*(12) = 945.25, *p* <.001, with Nagelkerke *R²* = .493.

Pornography-related symptom count showed a strong association with SIPA. Each additional symptom was associated with a more than twofold increase in the odds of identifying oneself as addicted, *OR* = 2.10, 95% CI [1.93, 2.28], *p* <.001. Frequency of pornography consumption was also strongly associated with self-identification. Each one-category increase in PUF corresponded to a more than twofold increase in the odds of self-identifying as addicted, *OR* = 2.14, 95% CI [1.84, 2.50], *p* <.001.

Biological sex was also significantly associated with SIPA: men were substantially more likely than women to consider themselves addicted to pornography, *OR* = 2.47, 95% CI [1.57, 3.89], *p* <.001. In contrast, place of residence, age, religiosity, ease of establishing personal relationships, sexual-life satisfaction, age at first exposure to pornography, perceived adequacy of sexual education, sexual disorders, and paraphilic disorder were not significantly associated with self-identification after controlling for the other variables included in the model (see **Table 2**).

Overall, SIPA was associated primarily with PUF and the accumulation of pornography-related symptoms, whereas most psychosocial and psychosexual variables showed no independent association.

### 3.4 Differences between concordance groups

Bivariate analyses revealed significant differences among the four concordance groups across several demographic, psychosocial, and psychosexual characteristics (**Table 3**).

**Table 3:** Characteristics of the four PWD–SIPA concordance groups.

Variable	Concordant non-problematic group	PWD-only group	SIPA-only group	Both PWD and SIPA group	Test
n	8,163	267	118	152	—
Biological sex (%)					*χ²* = 373.49, *p* <.001, *V* = .207
Male	31.8	58.8	83.9	81.6	
Female	68.2	41.2	16.1	18.4	
Place of residence, %					*χ²* = 6.39, *p* = .094, *V* = .027
Non-capital	91.2	95.1	93.2	93.4	
Capital	8.8	4.9	6.8	6.6	
Age, *M (SD)*	23.41 (6.35)	22.76 (5.14)	24.00 (7.89)	23.14 (5.26)	Welch *F* = 1.66, *p* = .176, *η²* <.001
Religiosity, *M (SD)*	1.98 (1.96)	2.28 (2.22)	1.79 (1.78)	2.28 (2.30)	Welch *F* = 2.84, *p* = .039, *η²* = .001
Ease of establishing personal relationships, *M (SD)*	7.15 (2.14)	6.63 (2.45)	6.85 (2.44)	6.61 (2.47)	Welch *F* = 6.80, *p* <.001, *η²* = .003
Satisfaction with sex life, *M (SD)*	6.74 (2.77)	5.73 (2.93)	6.24 (2.80)	5.56 (3.01)	Welch *F* = 15.24, *p* <.001, *η²* = .006
Age at first pornography exposure, *M (SD)*	14.01 (3.01)	12.78 (2.92)	12.19 (2.84)	12.51 (2.85)	Welch *F* = 43.01, *p* <.001, *η²* = .013
Adequate sexual education, %					*χ²* = 8.56, *p* = .036, *V* = .031
No	29.2	33.0	27.1	38.8	
Yes	70.8	67.0	72.9	61.2	
Sexual disorder, %					*χ²* = 10.11, *p* = .018, *V* = .034
No	81.6	79.4	92.4	82.9	
Yes	18.4	20.6	7.6	17.1	
Paraphilic disorder, %					*χ²* = 179.83, *p* <.001, *V* = .144
No	98.8	94.0	94.9	86.8	
Yes	1.2	6.0	5.1	13.2	

No significant age differences were observed between the groups, Welch’s *F* = 1.66, *p* = .176, η² <.001. Significant group differences emerged for religiosity, Welch’s *F* = 2.84, *p* = .039, η² = .001; ease of establishing personal relationships, Welch’s *F* = 6.80, *p* <.001, *η²* = .003; sexual-life satisfaction, Welch’s *F* = 15.24, *p* <.001, *η²* = .006; and age at first exposure to pornography, Welch’s *F* = 43.01, *p* <.001, *η²* = .013. Although these differences were statistically significant, all effect sizes were small according to conventional benchmarks.

The largest difference among the continuous variables concerned age at first exposure to pornography. Participants in the concordant non-problematic group reported the latest first exposure (*M* = 14.01 years), whereas all three groups characterized by symptom-defined PWD, SIPA, or both reported substantially earlier exposure (*M* = 12.19–12.78 years). Participants in the concordant non-problematic group also reported the highest level of sexual-life satisfaction (*M* = 6.74), whereas the group with both PWD and SIPA reported the lowest level (*M* = 5.56).

Significant associations were also found for biological sex, *χ²*(3) = 373.49, *p* <.001, Cramér’s *V* = .207; adequate sexual education, *χ²*(3) = 8.56, *p* = .036, Cramér’s *V* = .031; sexual disorders, *χ²*(3) = 10.11, *p* = .018, Cramér’s *V* = .034; and paraphilic disorder, *χ²*(3) = 179.83, *p* <.001, Cramér’s *V* = .144. In contrast, place of residence was not significantly associated with concordance-group membership, *χ²*(3) = 6.39, *p* = .094.

Paraphilic disorders also showed substantial group differences. Whereas only 1.2% of participants in the concordant non-problematic group met criteria for a paraphilic disorder, the prevalence increased to 6.0% in the PWD-only group, 5.1% in the SIPA-only group, and 13.2% in the group with both PWD and SIPA.

### 3.5 Multinomial logistic regression analysis of factors associated with concordance-group membership

To identify factors that simultaneously distinguished the four concordance groups, a multinomial logistic regression analysis was performed, with the concordant non-problematic group as the reference category (**Table 4**).

**Table 4:** Multinomial logistic regression analysis of factors associated with PWD–SIPA concordance group membership.

Variable	PWD-only group OR (95% CI)	SIPA-only group OR (95% CI)	Both PWD and SIPA group OR (95% CI)
PUF	1.88 [1.68, 2.12]***	2.97 [2.42, 3.63]***	2.75 [2.29, 3.30]***
Biological sex (Male vs Female)	1.21 [0.85, 1.71]	1.99 [1.09, 3.62]*	2.76 [1.59, 4.80]***
Place of residence (Capital vs Non-capital)	0.48 [0.25, 0.96]*	1.06 [0.50, 2.24]	0.67 [0.29, 1.56]
Age	0.98 [0.96, 1.01]	1.02 [0.99, 1.05]	0.99 [0.96, 1.02]
Religiosity	1.11 [1.04, 1.18]**	0.98 [0.87, 1.11]	1.11 [1.01, 1.22]*
Ease of establishing personal relationships	0.92 [0.87, 0.98]*	0.97 [0.89, 1.07]	0.93 [0.85, 1.01]
Satisfaction with sex life	0.95 [0.91, 1.00]	1.02 [0.94, 1.09]	0.96 [0.90, 1.03]
Age at first pornography exposure	0.94 [0.89, 0.99]*	0.98 [0.90, 1.06]	0.98 [0.91, 1.05]
Adequate sexual education (Present vs Absent)	0.77 [0.57, 1.03]	0.85 [0.54, 1.34]	0.54 [0.37, 0.80]**
Sexual disorder (Present vs Absent)	1.74 [1.21, 2.51]**	0.93 [0.42, 2.02]	2.16 [1.26, 3.72]**
Paraphilic disorder (Present vs Absent)	2.65 [1.40, 5.00]**	2.50 [1.01, 6.25]*	5.18 [2.68, 10.00]***

Reference group: Concordant non-problematic group. PWD, pornography-watching disorder; OR, odds ratio; CI, confidence interval. Model fit: *χ²*(33) = 858.44, *p* <.001; Nagelkerke *R²* = .241. *p <.05. **p <.01. ***p <.001.

The overall model was statistically significant, *χ²*(33) = 858.44, *p* <.001, with Nagelkerke *R²* = .241.

For the PWD-only group, higher PUF, greater religiosity, greater difficulty establishing personal relationships, earlier first exposure to pornography, sexual disorders, and paraphilic disorders significantly increased the likelihood of membership relative to the concordant non-problematic group. Lower sexual life satisfaction showed the same direction of association but did not reach conventional statistical significance (*p* = .051).

For the SIPA-only group, the pattern was notably different. Higher PUF and male sex were significant factors, whereas most psychosocial and psychosexual variables were not significantly associated with group membership. Paraphilic disorder showed a weak positive association, whereas religiosity, relationship formation, sexual-life satisfaction, age at first pornography exposure, and sexual disorders were unrelated to SIPA-only membership after adjustment for the other factors.

For the group with both PWD and SIPA, higher PUF, male sex, greater religiosity, inadequate sexual education, sexual disorders, and paraphilic disorders significantly increased the likelihood of belonging to this category.

Overall, the multinomial model demonstrated that individuals who perceived themselves as addicted despite not meeting symptom-based criteria differed substantially from participants with symptom-defined PWD. Whereas symptom-defined PWD was associated with a broader constellation of sociodemographic, psychosexual, and psychosocial correlates, SIPA-only membership was associated primarily with PUF and male sex.

### 3.6 Sensitivity analyses

Two sensitivity analyses were conducted to assess the robustness of the findings.

First, PUF was modeled as a continuous variable rather than as a categorical variable. This decision was based on preliminary analyses showing a highly monotonic relationship between PUF and both symptom-defined PWD and SIPA. Specifically, the proportion of participants reporting SIPA increased progressively across all frequency categories, ranging from 0.1% among those who had not viewed pornography during the previous year to 37.7% among those reporting pornography use at least five times per day. This was further supported by univariable binary logistic regression models including PUF as the sole predictor, which showed only negligible differences in explanatory power between the continuous and categorical specifications for both symptom-defined PWD and SIPA (Nagelkerke R² = .183 vs. .185 for PWD, and .277 vs .280 for SIPA). Therefore, the more parsimonious continuous specification was retained in the final models.

Second, because previous studies using the same database focused primarily on younger participants, all multivariable analyses were repeated in the subsample of participants aged 35 years or younger. The results were highly similar to those obtained in the full sample. For instance, in the model predicting SIPA, pornography-related symptoms, PUF, and biological sex remained significant factors, whereas age remained non-significant. Model fit was similar in the younger subsample (Nagelkerke *R²* = .501) and in the full sample (Nagelkerke *R²* = .493). These findings indicate that the observed associations were not driven by the inclusion of older participants and support the robustness of the main results within the younger subsample.

## 4 Discussion

The present study compared two commonly used operationalizations of problematic pornography involvement: the symptom-defined classification of PWD and SIPA, rather than two formally established diagnostic entities. Three main findings emerged. First, symptom-defined PWD and SIPA showed only partial overlap. Second, the two constructs displayed markedly different patterns of correlates. Third, multinomial analyses demonstrated that individuals who identified themselves as addicted despite not meeting symptom-based criteria differed from those meeting symptom-defined PWD criteria. Together, these findings indicate that the two operationalizations overlap only partially and identify somewhat different subsets of individuals (5, 9, 10, 13, 18). Because the data are cross-sectional, the regression findings are interpreted as statistical associations and should not be taken as evidence of causal or temporal relationships.

The observed prevalence rates further support this conclusion. While 4.8% of participants met criteria for symptom-defined PWD, only 3.1% considered themselves addicted to pornography. Moreover, agreement between the two classifications was moderate rather than strong. A substantial proportion of individuals who met symptom-based criteria did not identify themselves as addicted, whereas a considerable proportion of those who self-identified as addicted did not meet symptom-based criteria. These findings are consistent with previous research indicating that direct self-identification as addicted does not necessarily correspond to checklist- or scale-based indicators of PPU and may be influenced by additional psychological, moral, or social factors (5, 14, 20, 21). They also support concerns that prevalence estimates may vary considerably depending on whether problematic pornography involvement is defined through symptom-based thresholds, single-item self-identification, or broader self-perceived problems (5, 13, 20).

Consistent with earlier work, higher PUF was a strong factor associated with PWD, but frequency alone did not fully account for symptom-defined problems (18). This is also in line with broader evidence showing that high-frequency pornography use is not necessarily problematic unless accompanied by impaired control, distress, or functional impairment (9). In the present study, PWD was also associated with greater religiosity, more difficulties establishing personal relationships, lower sexual-life satisfaction, earlier exposure to pornography, self-reported sexual disorder status, and self-reported paraphilic disorder status. These findings indicate that the checklist-based PWD classification was associated with a broader range of sociodemographic, psychosexual, and psychosocial variables than PUF alone.

The association between PWD and religiosity, as well as the absence of an independent association between religiosity and self-identification, should be interpreted cautiously. Previous studies have often linked self-perceived pornography addiction to religiosity, moral disapproval, and moral incongruence, in addition to PUF (5, 14, 20, 21). However, the present study measured religiosity as adherence to religious norms rather than directly assessing moral disapproval of pornography or moral incongruence. Therefore, the findings should not be interpreted as evidence against processes of moral incongruence. Rather, they suggest that religiosity alone may be an incomplete proxy for the moral, normative, and subjective appraisal mechanisms through which pornography use is interpreted and experienced (5, 10, 21). Future studies including direct measures of moral incongruence will be needed to evaluate this possibility.

The observed associations with sexual-life satisfaction, relationship difficulties, and self-reported sexual disorder status are consistent with previous reports linking symptom-based problematic pornography involvement to broader psychosocial and sexual functioning (11, 15, 18). Compared with self-identification, these variables showed a more consistent association with the symptom-based classification.

In contrast, the pattern observed for SIPA was considerably more restricted. Self-identification was strongly associated with PUF and pornography-related symptom burden, and men were significantly more likely than women to consider themselves addicted. However, most sociodemographic, psychosexual, and psychosocial variables that predicted symptom-defined PWD were not independently associated with self-identification after adjustment for the other factors. Variables such as relationship difficulties, sexual-life satisfaction, age at first exposure to pornography, sexual disorders, and place of residence did not contribute significantly to explaining who perceived themselves as addicted. These findings suggest that direct self-identification may reflect how individuals evaluate and label their pornography-related experiences more than the broader psychosocial and psychosexual profile associated with symptom-defined PWD. However, the present study did not directly assess the appraisal processes underlying self-identification. Future research should therefore examine whether moral incongruence, perceived loss of control, stigma, or other interpretive processes help explain why some individuals adopt an addiction label without meeting the symptom-based threshold.

The multinomial analyses provide particularly important insight into the distinction between the two constructs. Participants who met the study-defined PWD threshold but did not identify themselves as addicted showed a profile broadly consistent with higher symptom endorsement, including associations with religiosity, interpersonal difficulties, lower sexual-life satisfaction, earlier exposure to pornography, sexual disorders, and paraphilic disorders. By contrast, individuals who identified themselves as addicted despite not meeting symptom-based criteria were distinguished primarily by higher PUF and male sex, while most psychosocial and psychosexual variables were unrelated to group membership. Notably, this SIPA-only group appeared considerably closer to the concordant non-problematic group than to the symptom-defined PWD groups on most measured characteristics. These findings suggest that the discordant profiles reflect more than simple measurement disagreement and that symptom-defined PWD and SIPA may capture partly different dimensions of problematic pornography involvement. The two discordant groups may also reflect different appraisal processes. Individuals in the SIPA-only group may attach an addiction label to frequent use or to symptoms that remain below the selected threshold, whereas individuals in the PWD-only group may report several symptoms without interpreting or labeling their behavior as an addiction. These possibilities cannot be tested with the present data, but they help explain why relying on only one operationalization may provide an incomplete picture of pornography-related difficulties.

From a conceptual perspective, the present findings suggest that neither direct self-identification nor checklist-based classification should automatically be regarded as a proxy for the other. Both operationalizations rely on self-reported information, but they organize that information differently. The PWD classification aggregates responses across multiple symptom indicators, whereas SIPA captures whether participants adopt an addiction label in response to a direct question. In the present study, the checklist-based PWD classification was associated with a broader range of sociodemographic, psychosexual, and psychosocial characteristics, whereas SIPA was more closely associated with PUF and pornography-related symptom burden. These differences do not establish that one approach is inherently more objective or valid; rather, they indicate that the two approaches capture partly distinct aspects of participants’ self-reported pornography-related experiences.

Consequently, studies relying exclusively on self-identification may not assess the same underlying construct as studies employing symptom-based measures. This distinction is clinically relevant because compulsive sexual behavior disorder in ICD-11 emphasizes persistent failure to control sexual impulses or behaviors resulting in distress or impairment, while also cautioning that distress based solely on moral judgments or disapproval is insufficient for diagnosis (7, 8). Although PWD is not a formal DSM-5 diagnosis, the present results support the broader principle that clinical assessment should integrate multiple sources of self-reported information rather than relying exclusively on either a symptom checklist or an addiction self-label.

Several limitations should be acknowledged. First, the cross-sectional design precludes causal interpretations regarding the observed associations. It is therefore not possible to determine whether factors such as sexual dissatisfaction, relationship difficulties, or earlier exposure contribute to PWD, result from PWD, or reflect shared underlying vulnerabilities. Second, both PWD and SIPA were derived entirely from self-reported information and may therefore be influenced by recall bias, social desirability, symptom interpretation, and self-presentation. The two operationalizations differ in format—a structured symptom checklist versus a direct self-identification item—but neither constitutes an objective or clinically verified assessment. In addition, because the SIPA item was administered immediately after the symptom checklist, responses to the self-identification item may have been influenced by prior consideration of the symptom indicators. A question-order or priming effect therefore cannot be excluded. Future studies should examine whether the observed concordance between the two operationalizations remains similar when the order of the symptom checklist and the self-identification item is varied. The survey also did not provide a specific definition of pornography, and participants may therefore have differed in what they considered pornographic material. Different forms or sources of pornography use should be examined separately in future research. Third, although the sample was large, it was drawn from Hungary and was recruited online, which may limit the generalizability of the findings to other cultural contexts and to less internet-active populations. Cross-cultural research suggests that associations among pornography use, religiosity, moral incongruence, and self-perceived addiction may vary across social and cultural contexts, even when similar mechanisms are observed across countries (22, 23). Fourth, symptom-defined PWD was operationalized using a DSM-5-inspired symptom framework rather than clinical assessment, and therefore should not be interpreted as equivalent to a formal clinical diagnosis. Fifth, particular caution is warranted when interpreting the self-reported sexual disorder and paraphilic disorder variables. Although these variables were derived using DSM-5-based self-report criteria, they were not established through structured clinical interviews or professional diagnostic assessment. Because they concern highly sensitive aspects of sexuality, responses may have been influenced by social desirability, shame, deliberate concealment or underreporting, limited self-knowledge, and differences in the interpretation of questionnaire items. These factors may have introduced classification error. Consequently, the variables should be interpreted as self-reported indicators rather than confirmed clinical diagnoses. Sixth, the study did not include a direct measure of moral incongruence or moral disapproval of pornography use, limiting the ability to test whether self-identification without symptom-defined PWD reflects moral conflict, subjective distress, perceived loss of control, or other appraisal processes. Seventh, although the survey included several questions on lifetime sexual abuse, it did not include a broader assessment of trauma, adverse childhood experiences, psychiatric comorbidities, domestic violence, family and peer support, housing stability, or general quality of life. Future studies should examine how these broader psychosocial domains, together with specific trauma-related experiences such as sexual abuse, contribute to concordant and discordant PWD and SIPA profiles.

**Figure 2:**
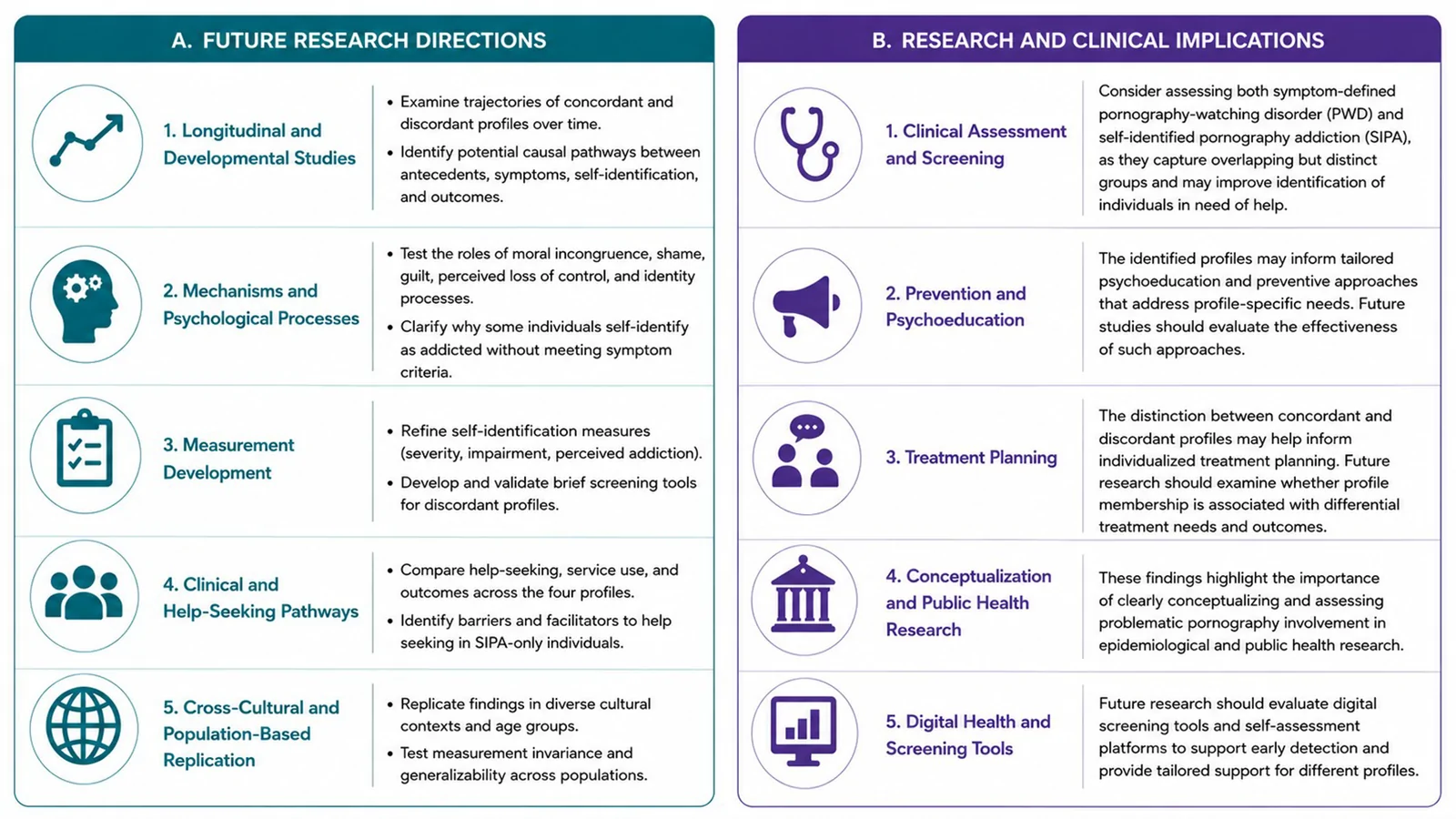
Future research directions and research and clinical implications of the present findings.

Despite these limitations, the present study contributes to ongoing debates regarding the conceptualization and assessment of problematic pornography involvement by directly comparing two commonly used operationalizations within the same sample. Future research should continue to investigate the mechanisms underlying self-identification as addicted by incorporating direct measures of moral incongruence and moral disapproval, and by examining how subjective perceptions of addiction relate to symptom severity, psychological distress, sexual functioning, help-seeking behavior, and the cognitive processes involved in interpreting pornography-related experiences. Several promising directions, together with potential research and clinical implications of the present findings, are summarized in **Figure 2**. Overall, these directions emphasize the need for future studies to distinguish more clearly between symptom-defined PWD and self-identified addiction, to investigate the mechanisms underlying concordant and discordant classifications, and to evaluate how these different operationalizations may inform assessment and intervention in both research and clinical settings. Longitudinal and clinically validated studies will be particularly important for determining whether concordant and discordant profiles represent different developmental pathways, distinct underlying mechanisms, or qualitatively different forms of problematic pornography involvement. Overall, the findings indicate that symptom-based classification and self-identification represent complementary rather than interchangeable operationalizations of problematic pornography involvement.

## Data Availability

The original contributions presented in the study are included in the article/supplementary material. Further inquiries can be directed to the corresponding author.
